# Classifying dermoscopic patterns of naevi in a case-control study of melanoma

**DOI:** 10.1371/journal.pone.0186647

**Published:** 2017-10-17

**Authors:** Seamus R. McWhirter, David L. Duffy, Katie J. Lee, Glen Wimberley, Philip McClenahan, Natalie Ling, Marco Ardigo, Helmut Schaider, H. Peter Soyer, Richard A. Sturm

**Affiliations:** 1 Dermatology Research Centre, The University of Queensland Diamantina Institute, Translational Research Institute, Brisbane, Australia; 2 QIMR Berghofer Medical Research Institute, Brisbane, Australia; 3 San Gallicano Dermatological Institute, IRCCS, IFO, Rome, Italy; 4 Department of Dermatology, Princess Alexandra Hospital, Brisbane, Australia; Universidade de Sao Paulo, BRAZIL

## Abstract

Changes in dermoscopic patterns of naevi may be associated with melanoma; however, there is no consensus on which dermoscopic classification system is optimal. To determine whether different classification systems give comparable results and can be combined for analysis, we applied two systems to a case-control study of melanoma with 1037 participants: 573 classified using a “1/3 major feature” system, 464 classified based on rules of appearance, and 263 classified with both criteria. There was strong correlation for non-specific (Spearman R = 0.96) and reticular (Spearman R = 0.82) naevi, with a slight bias for globular naevi with the rules of appearance system. Inter-observer reliability was high for the rules of appearance system, particularly for reticular naevi (Pearson >0.97). We show that different classification systems for naevi can be combined for data analysis, and describe a method for determining what adjustments may need to be applied to combine data sets.

## Introduction

Dermoscopy is a technique developed in the 1950s for the clinical evaluation of skin lesions, which was widely adopted by Dermatologists and, to a lesser extent, General Practitioners in the late 1980s [1, 2]. Its utility in allowing users to differentiate cancerous from non-cancerous lesions has been well documented [3], and now its relevance is being tested in a number of epidemiological studies into melanoma risk. Despite this, a consensus classification system for the dermoscopic appearances of structures seen using this tool is lacking, although several have been proposed [4–9]. Not only do different studies employ distinctive classification systems, but inter- and intra-observer agreement on class in any given system is far from perfect, even in experts in dermoscopy [4]. The purpose of this study was to test whether the results from two different naevus classification systems applied by researchers in the ongoing Brisbane Naevus Morphology Study (BNMS) [10, 11] could be combined for analysis. This question has important implications for studies into naevi, as classification systems may evolve or be refined in the future, and the process of reclassifying naevi is time consuming when dermoscopic images of all naevi ≥5mm in diameter are analysed on all body sites, as is being conducted in the BNMS.

**Fig 1:**
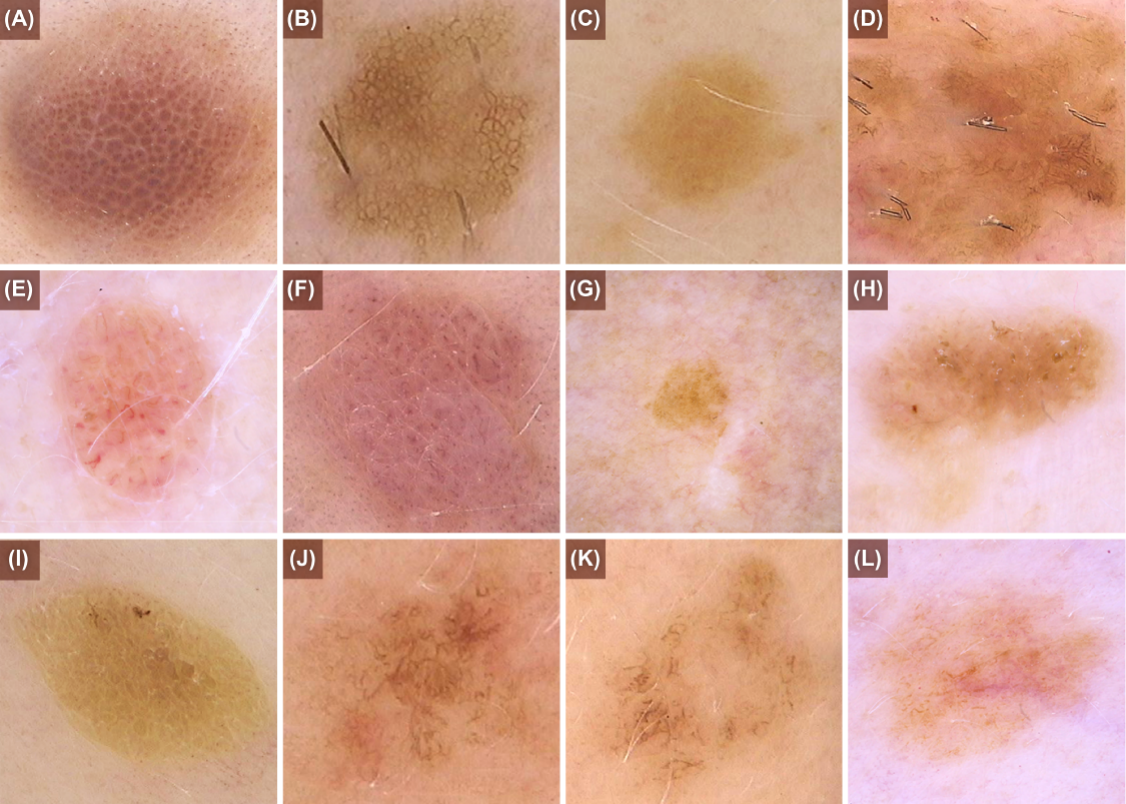
Examples of naevi in the new BNMS classification groups: (a) globular, (b) reticular, (c) homogenous and (d) complex. Examples of structures that could be mistaken for globules: (e, f) red dots are blood vessels, not pigment globules, (g) dots are too small to be globules and (h, i) yellow structures are keratin, not pigment globules. Examples of structures that could be mistaken for reticular: (j, k) lines do not form a complete net, so these naevi are complex not reticular; (l) chaotic appearance with widespread structures that are not reticular or globules.

Broadly, naevi can be classified as either congenital, being present at birth or arising within the first two years of life, or acquired, presenting at a later stage [12]. Although subsets of naevi exist within both classifications, such as Reed, Spitz and blue naevi, these can all be primarily categorized by their pigment pattern as viewed by dermoscopy (see Fig 1). The first reported application of a dermoscopic classification system to a formal study was performed by Hofmann-Wellenhoff *et al* in 2001, who categorized naevi into reticular, globular, homogenous, or combinations of the three, with those not fitting any category labelled as unclassified [5]. This seven-category system was applied to a population with clinically determined dysplastic naevi; however Gamo *et al* (2013) applied the same system to a population with all types of acquired naevi [13].

A simplified model of this seven-category system was developed by Lipoff *et al* (2008) when 20 individuals with a history of melanoma were compared with controls with a high number of atypical/dysplastic naevi to determine if certain dermoscopic patterns were related to melanoma risk [6]. The criteria were adapted from Hofmann-Wellenhoff [5], with naevi exhibiting combinations of patterns grouped as ‘complex’, unless the secondary pattern was homogenous. This created four categories: Globular, Reticular, Homogenous or Complex. Lipoff *et al* stipulated that three or more globules must be present for the globular type, and the network could be diffuse or patchy for the reticular type [6]. Fonseca *et al* applied and tested the same classification system as Scope *et al* when looking into the anatomical distribution of dermoscopic patterns [9, 14]. The Fonseca study method defined a globule as a well demarcated structure, round-to-oval, greater than >0.1mm in diameter [9].

Individual risk of developing melanoma is strongly correlated with total number of acquired naevi present on the skin. The BNMS is particularly aimed at addressing the question as to whether counts of naevi of any particular dermoscopic class are a better indicator of melanoma risk than overall count. Given the evolution in dermoscopic classification systems throughout the recruitment and implementation of the BNMS, a system similar to that used by Scope *et al* [14] was applied for the first 573 participants, followed by a system modified from Fonseca *et al* [9] for 464 participants, where 263 individuals scored using both systems. The present paper concentrates on whether body counts of naevi from these different systems can be safely combined to answer our epidemiological hypotheses. We conclude that it is possible to combine results from the two systems for meaningful analysis.

## Materials and methods

This study was approved by the Human Research Ethics Committee of Princess Alexandra Hospital and The University of Queensland and conducted in accordance with the Declaration of Helsinki. Participants provided written consent after receiving a Participant Information and Consent Form. Participants under 18 years of age also required the written consent of a parent or guardian.

A suitable dermoscopic classification system was developed to apply to the BNMS, an ongoing epidemiological study of naevus and melanoma genes in participants recruited from South-East Queensland [10, 11]. Participants were recruited between October 2009 and March 2016 from the Melanoma Unit and Dermatology Department of the Princess Alexandra Hospital (Brisbane, Australia), private dermatology clinics, the Brisbane Twin Naevus Study, and QSkin participants [15]. Participants were required to be over the age of 15 years, and there was no upper age limit. Case participants were those who had a personal history of one or more primary melanomas, verified by the referring dermatologist or attendance at the Melanoma Unit; control participants were those who had no personal history of melanoma, as reported by the participant, and were predominantly drawn from the Brisbane Twin Naevus Study. Following a 60-participant pilot conducted from October 2009 to March 2010, it was determined that a maximum of 100 case and 100 control patients could be imaged per year. The study length of 6.5 years (from October 2009 to March 2016) was determined by funding constraints.

All participants had naevi counts and dermoscopic imaging performed by a trained nurse or research assistant on all naevi ≥5mm in diameter. Sixteen regions were observed for naevi, covering the entire body except areas covered by underwear, the scalp and the soles of the feet. A body image map was generated by the Vectra® WB360 3D body imager (Canfield Scientific Inc., NJ USA) or FotoFinder® (Bad Birnbach, Germany) to aid the cataloguing of naevi in individuals. A digital dermoscope was used to capture dermoscopic images, which were then uploaded onto the FotoFinder® or Vectra® software systems. One of three research assistants then allocated each image to a dermoscopic subclass. The total counts for each dermoscopic subclass were tallied for each participant, as well as the total number of naevi ≥5mm. Participants with no naevi ≥5mm were included and assigned a value of 0 in all categories.

### The old BNMS classification system

The first classification system classified all naevi by their predominant dermoscopic pattern, which had to be present over at least one-third of the lesion’s area. The categories were globular (round to oval structures), reticular (net-like structures) or non-specific (which included homogenous naevi and those that had another pattern which was not considered either globular or reticular). If no pattern occupied more than one-third of the lesion’s area, the lesion was considered non-specific.

### The new BNMS classification system

Following the publication by Fonseca *et al* [9], revision of the existing classification system was performed and an expert Dermatologist (H.P.S) formulated a new system. This classification system was termed the new BNMS classification system and was applied to 210 new participants recruited to the study and 263 participants who had already been classified with the old BNMS classification system.

The naevi were assessed for dermoscopic patterns and grouped into four classes. (1) Primarily reticular: a distinct pigment network is present with <3 globules. There must be a complete net, not just open lines. (2) Primarily globular: ≥3 globules are present without a pigment network. Globules are defined as symmetric, round to oval, well demarcated structures with a diameter >0.1mm. Red and yellowish structures are not included as these represent vascular and keratin structures respectively. (3) Homogenous: tan, brown, blue, or pink structureless lesion with neither a pigment network nor ≥3 globules present. (4) Complex: (a) both network and ≥3 globules are present, with or without structureless areas OR (b) chaotic with >2 colours present or has widespread structures that are neither reticular nor globular OR (c) a distinct peripheral rim of globules or starburst pattern is present, with or without other structures or structureless areas. See Fig 1 for examples of dermoscopic patterns and their classification group and Table 1 for a comparison of the old and new systems.

**Table 1:** Dermoscopic pattern criteria of the old BNMS classification system and new BNMS classification system.

	Old BNMS System	New BNMS System
Reticular	• At least 1/3 of the lesion is covered by a reticular network	• Distinct pigment network, in a complete net not open lines • <3 globules
Globular	• At least 1/3 of the lesion is covered by globules	• ≥3 globules: round to oval, well demarcated structures, diameter >0.1mm • Red or yellow structures are vascular or keratin structures, not globules
Non-specific	• The lesion does not meet the criteria for either reticular or globular naevi	• This category has been divided into homogenous and complex categories
Homogenous	NA	• Tan, brown, blue, or pink structureless lesion • No reticular network • <3 globules
Complex	NA	• Both network and ≥3 globules • OR chaotic with >2 colours or has widespread structures that are neither globular nor reticular • OR a distinct peripheral ring of globules or a starburst pattern, with or without other structures

Three Dermatologists (H.P.S, H.S. and M.A.) classified 554 naevi in a subset of 20 participants in order to refine the rules of the new BNMS system. These Dermatologists trained three research assistants to apply the new criteria to the dermoscopic images of the BNMS. The research assistants also classified the subset of 20 participants separately, and were tested for inter-observer reliability by calculating a Pearson correlation coefficient.

### Statistics

The R program was used to analyse data from the 263 participants who had been classified by both classification systems. We cross-classified individual naevi to assess agreement between systems, as well as comparing total counts of naevi of comparable classes on each individual. Scatterplots were constructed for each dermoscopic subclass count, with lines of best fit for a linear model compared with the lines of identity. Spearman’s rank correlation coefficients were calculated for each subclass as a method of determining consistency between the systems, since naevus counts are non-Gaussian in distribution. We also calculated Pearson correlations for log-transformed counts (actually N+1), as this transformation was that best supported by Box-Cox regression results. To compare the reliability of systems in allocating non-specific naevi, homogenous and complex counts were combined for the new BNMS system.

## Results

A total of 24 243 naevi ≥5mm in 1032 participants were imaged and were assigned a dermoscopic pattern. 573 participants were classified using the old BNMS system and 210 using the new BNMS system; both systems were applied to a further 263 participants. Potential participants who were invited but declined participation gave one or more of the following reasons: time constraints; unable to come to the hospital for imaging; did not wish to undergo full-body imaging; was too unwell to stand up long enough for the imaging process; was not interested in participating in research.

The participants were 50.2% (518) male with a median age of 46 years. 47.7% (493) were cases and 52.2% (539) were control participants. Case participants had a mean of 34.3 naevi; control participants had a mean of 13.7 naevi ≥5mm.

The results from the inter-observer reliability exercise, which involved three investigators applying the new BNMS classification system prior to the study, is included in Tables 2 and 3. Inter-observer reliability was highest for reticular naevi, where it was very strong (Pearson >0.97), and lowest for complex naevi (Pearson >0.7). No two investigators showed consistently stronger correlating decisions.

**Table 2:** Rater agreement calculated as m-rater Kappa coefficients for each class, and overall agreement.

	Kappa	Std error	Z	*P* value
Globular	0.497	0.028	17.76	<0.001
Reticular	0.669	0.028	23.93	<0.001
Homogenous	0.561	0.028	20.07	<0.001
Complex	0.402	0.028	14.36	<0.001
Overall	0.526	0.017	30.84	<0.001

**Table 3:** Pearson correlation coefficients of 3 observers for counts of naevi of different dermoscopic patterns in 20 participants.

	Globular	Reticular	Homogenous	Complex
RA1 vs RA2	0.83	0.99	0.88	0.87
RA1 vs RA3	0.89	0.97	0.93	0.75
RA2 vs RA3	0.88	0.97	0.87	0.70

RA: research assistant

**Fig 2:**
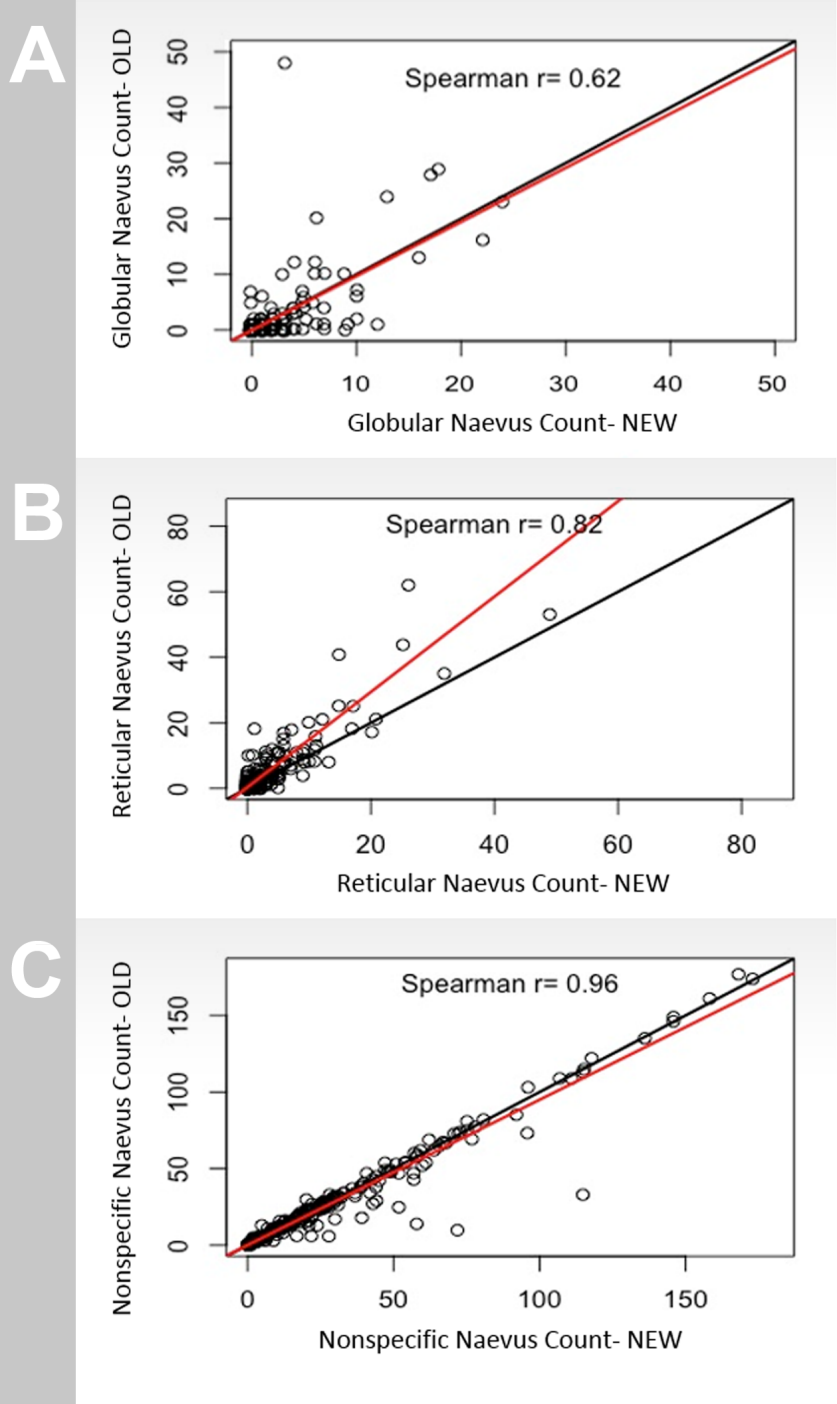
Correlation of number of dermoscopic naevi subtypes counted for individuals by the old BNMS classification system (Y axis) and new BNMS classification system (X axis) for (a) globular, (b) reticular and (c) nonspecific naevi.

Morphological subclasses of the 263 participants classified with both systems were compared for systematic bias between the two classification systems by inspection of scatterplots (Fig 2). The new BNMS classification system did not show a specific bias for globular naevi, with values lying evenly either side of the line of identity. The correlation of the systems was good (Spearman’s r = 0.62) for this subclass (Fig 2A). A slight bias for under-calling reticular naevi was found with the new BNMS classification system (Fig 2B), while there was an improved correlation for this subtype (r = 0.82). Correlation between classification systems was closest to perfect for non-specific counts (r = 0.96) (Fig 2C). This was performed by comparing the number of non-specific naevi from the old system with the combined total of homogenous and complex naevi from the new BNMS system.

## Discussion

We compared two different classification systems (Table 1) to decide whether dermoscopic patterns assigned to a large database of melanoma cases and controls, using two different systems, could be combined for analysis. Consistency between the two classification systems varied according to the dermoscopic subclass, which was to be expected due to the more detailed criteria stipulated in the new system. One of the most significant findings was that combining the number of homogenous and complex naevi correlated very well with the number of non-specific naevi. This means that the decision to segregate non-specific naevi into more specific subtypes is justified, and will enable grouping of the two BNMS classification systems for the purpose of further data analysis and comparison to other data sets.

While Cohen kappa values for agreement about the dermoscopic subtype of any individual naevus are moderate (0.53 overall, Table 2), the agreement for counts of each subtype in individual participants are moderate to high (Table 3) because the kappa values for counts of subtypes represent the sum of random variables. Prior studies of the epidemiology of naevi and how they relate to melanoma risk has concentrated on total number of naevi [16], and it is likely that total number of naevi in each dermoscopic subclass will also be the most useful measure to study.

**Fig 3:**
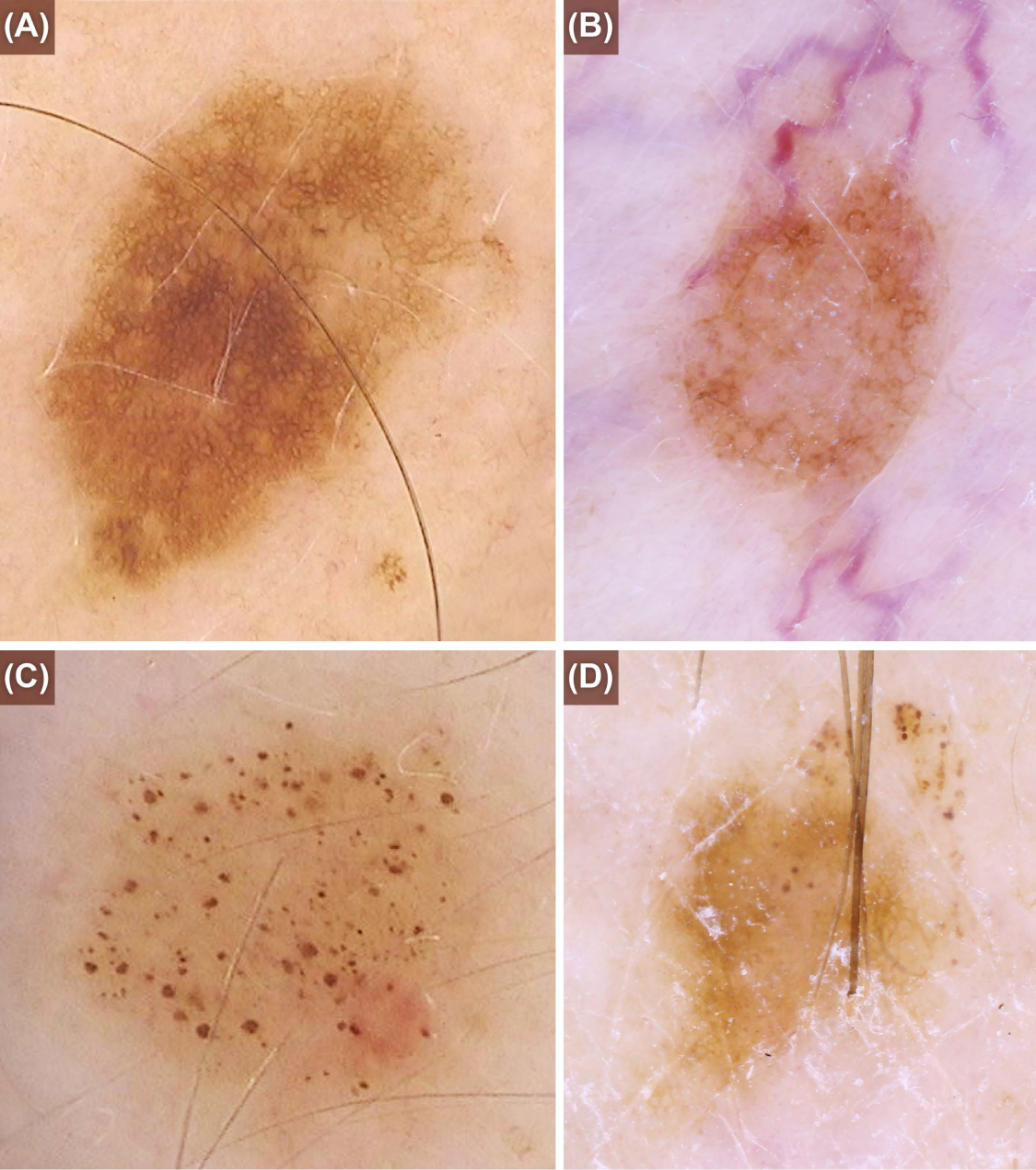
Differences in old and new BNMS criteria for reticular and globular patterns. Reticular: (a) the new system specifies that lines must create a complete net; (b) lines that form open shapes do not meet the criteria. Globular: (c) globules must be present over 1/3 or more of the naevus under the old BNMS system; (d) under the new BNMS system, 3 or more globules must be present but the globules can be confined to a relatively small area.

Systematic bias between the classification systems was seen for the reticular subtype. Slightly fewer reticular naevi were allocated under the new classification system, most likely because of the new BNMS classification system requiring a ‘complete net’ to satisfy the reticular category (Fig 3). This result will be accounted for in any further analysis of the dermoscopic patterns in future studies. The newer system also classified fewer naevi as globular, however the distribution of variation was true to the line of identity. This change was anticipated, as the new system excluded ‘vascular-like’ and keratin structures from the globular subtype. The reason for some participants having more globular naevi under the new system was most likely due to the number of naevi with three or more globules, which did not satisfy the criteria of occupying 1/3 of the lesion in the previous method (Fig 3).

By examining scatterplots plots (Fig 2), outliers were identified to be able to determine reasons for the discrepancies between the classification systems. A number of these were later found to have a melanoma gene of interest, *MITF* variant E318K, a gene previously associated with the reticular subtype of naevi [11]. As further gene analysis is performed on the data set, other naevus genes may be responsible for discrepancies in classifications, and hence the application of two different classification systems may be a novel method to determine naevus genes by testing for outliers.

The new BNMS classification system was observed to have very good consistency between the three investigators. Few studies which have applied dermoscopic classification systems in the past have tested for inter and intra-observer reliability. Fonseca *et al* [9] employed a system whereby two trained observers catalogued each naevus, as opposed to the single researcher in our method. Agreement between the two reviewers was assessed on 302 lesions and was high (K = 0.77) for their classification system. This supported the decision for the Fonseca system being incorporated as the foundation of the current system that was developed by the investigators. Providing more detailed criteria in our own new BNMS classification system may have improved the strong correlations.

A study published by Stanganelli *et al* (1995) assessed the dermoscopic patterns of 150 pigmented lesions for intra-observer reliability [17]. In labelling whether the reticular or globular pattern was present or not, K values were excellent (1.0) and good (0.67), respectively. When the investigator had to allocate one of six possible patterns to lesions, intra-observer agreement was also excellent (0.9). Other factors, such as whether patterns were discrete/prominent/regular/irregular, had poor agreement (<0.4) This illustrates that assigning descriptors of the dermoscopic pattern of pigmented lesions is far more reproducible than attempting to quantify the distributions of such patterns within a lesion, which our previous 1/3 BNMS classification system attempted to incorporate.

A study of dermoscopic classification of histopathologically confirmed pigmented lesions highlights that disparities do, however, exist in classifying naevi, even between those considered experts in this field [4]. In determining global dermoscopic patterns, the inter-observer K value among 40 international participants was fair (0.43). Again, certain patterns, such as pigment networks, were agreed upon more than other features, such as dots/globules, similar to our own study. One reason for the poor inter-observer reliability for dermoscopy may be that many studies have examined participants known to have many dysplastic naevi. These naevi are difficult to characterize by definition, with even histopathological consensus of these equivocal lesions being less than perfect, as found in a study by Ferrara *et al* [18].

A limitation of any study into dermoscopic subclasses of naevi is that inter-observer agreement will never be perfect; however, this makes the strong agreement within this new BNMS classification system all the more encouraging. This is the first known study to date to apply two different dermoscopic classification systems to the same data-set to compare models. We have shown a statistical method to allow for data called by different classification systems to be analysed together, which is important, as no real consensus on dermoscopic pattern classification currently exists, and studies such as the BNMS may run for long enough for new classification systems to be applied. It also provides a method for combining results from other naevus data-sets for meta-analysis or systematic reviews.
